# Evaluation of culture conditions for osteoclastogenesis in RAW264.7 cells

**DOI:** 10.1371/journal.pone.0277871

**Published:** 2022-11-17

**Authors:** Yin Cheng, Haixia Liu, Jing Li, Yujie Ma, Changheng Song, Yuhan Wang, Pei Li, Yanjing Chen, Zhiguo Zhang

**Affiliations:** 1 Institute of Basic Theory, China Academy of Chinese Medical Sciences, Beijing, China; 2 Institute of Basic Medical Sciences Chinese Academy of Medical Sciences, School of Basic Medicine Peking Union Medical College, Center of Excellence in Tissue Engineering, Chinese Academy of Medical Sciences, Beijing Key Laboratory of New Drug Development and Clinical Trial of Stem Cell Therapy (BZ0381), Beijing, China; BMSCE: BMS College of Engineering, INDIA

## Abstract

Osteoclasts are the only multinucleated cells *in vivo* responsible for bone resorption and are vital for regulating bone remodeling and maintaining bone mass. The RAW264.7 cell line is widely used to study osteoclastic differentiation and biological molecular mechanism. However, protocols for inducing osteoclast formation in RAW264.7 cells vary considerably between laboratories, hindering the replication of results. Therefore, we tested the influence of culture conditions on osteoclast differentiation, including cell density and receptor activator of nuclear factor kappa-B ligand (RANKL) concentrations with or without macrophage colony-stimulating factors (M-CSF). Tartrate-resistant acid phosphatase (TRAP) staining was used to detect the morphology of osteoclasts. qPCR was used to detect gene expression of osteoclast-specific gene marker cathepsin K (CTSK), osteoclast transcription factors c-Fos and nuclear factor of activated T cells, cytoplasmic 1 (NFATc1). The bone resorption function was evaluated by a scanning electron microscope (SEM). RANKL treatment increased multinucleated osteoclasts formation and increased CTSK, c-Fos and NFATc1 gene expression. Compared with RANKL treatment, M-CSF significantly decreased multinucleated osteoclasts formation, reduced CTSK gene expression and had little effect on c-Fos and NFATc1 gene expression. Concerning bone resorption activity, RANKL treatment increased bone resorption pits on bovine bone slices. Significantly higher levels of osteoclastogenesis were observed with RAW264.7-cell density of 2×10^4^ cells/well in 24-well plates. Our results suggest that the addition of 50 ng/ml M-CSF has no positive effect on osteoclastogenesis. RANKL treatment and cell density contribute to osteoclast formation, and the optimal conditions are beneficial when exploring osteoclast function and mechanism.

## Introduction

Osteoclasts originate from the monocyte/macrophage lineage and are unique multinucleated giant cells capable of bone resorption. They play indispensable roles in regulating bone remodeling and maintaining bone mass [1]. Increased or decreased osteoclast development or function results in an imbalance between bone formation and resorption, which can result in metabolic bone disorders such as osteosclerosis, osteolysis, and osteoporosis [2, 3]. As a result, it is critical to establish an *in vitro* culture system to further explore the biological properties of osteoclasts.

Osteoclasts are highly metabolized terminally differentiated cells. They are difficult to culture separately because of the small quantity, large volume and proximity to bone [4]. Therefore, the establishment of an efficient *in vitro* osteoclasts culture method has always been a research hotspot in the medical field. In 1982, British scientists Chambers and Magnus isolated and cultured mature osteoclasts from the limb bones of lactating rabbits for the first time, which laid a technical foundation for the mechanical isolation and culture of osteoclasts *in vitro* [5]. Since then, osteoclasts have been induced from primary cells and osteoclast precursor cell lines, including bone marrow macrophages, peripheral blood monocytes and splenocytes [6–8], as well as RAW264.7 cells.

The RAW264.7 macrophage cell line is widely applied in osteoclast precursor cells research. However, due to its strong adhesion, difficult digestion, sensitivity and high differentiation [9], the induction results vary between research laboratories and experimental approaches. Multiple induction methods have been reported, including receptor activator of nuclear factor kappa-B ligand (RANKL) [10, 11], RANKL and macrophage colony-stimulating factors (M-CSF) [12, 13], lipopolysaccharide [14, 15] and tumor necrosis factor alpha [16]. The first two methods are most commonly used. This study aimed to investigate whether RANKL and M-CSF were essential cytokines for osteoclast differentiation and the effect of cell density on induction efficiency in RAW264.7 cells. This research lays down a foundation for future studies of osteoclast function and biological molecular mechanisms.

## Materials and methods

### Cell line and reagents

RAW264.7 cells were bought from Cell Resource Center, Peking Union Medical College (Beijing, China). Recombinant Mouse TRANCE/RANKL/TNFSF11 (Cat#462-TEC-010) and Recombinant Mouse M-CSF Protein (Cat#416-ML-010) were purchased from R&D Systems (Minneapolis, MN). Dulbecco’s modified Eagle’s medium (DMEM, Cat#C12430500BT) with high glucose and fetal bovine serum (FBS, Cat#10099–141) were bought from Gibco (Grand Island, NY). Tartrate-resistant acid phosphatase (TRAP) stain kit (Cat#G1492) was purchased from Beijing Solarbio Science & Technology Co., Ltd (Beijing, China).

### Cell culture

RAW264.7 cells were routinely maintained in DMEM supplemented with 10% FBS at 37°C in a 5% CO_2_ humidified incubator, changing medium every other day. Cells at 80%-90% confluence were harvested with a pipette and passaged at a 1:3 ratio.

### TRAP staining

Different cell densities (5×10^3^ cells/well, 1×10^4^ cells/well, 2×10^4^ cells/well and 4×10^4^ cells/well) of RAW264.7 cells were plated into a 24-well plate and differentiated into osteoclasts in the presence of RANKL (50 ng/ml and 100 ng/ml) alone or with 50 ng/ml M-SCF. The induction medium was replaced every other day from day 0 to day 5. To detect multinucleated osteoclasts, TRAP staining was performed following the manufacturer’s instructions.

### Real-time quantitative polymerase chain reaction (qPCR)

On day 3, total RNA was isolated from cells with Trizol, cDNA was obtained by reverse transcription, and gene expression levels were detected by qPCR. The following primers were used: cathepsin K (*CTSK*)-forward 5´- CTCGGCGTTTAATTTGGGAGA-3´ and *CTSK*-reverse 5´- TCGAGAGGGAGGTATTCTGAGT-3´, *c-Fos*-forward 5´-CGGGTTTCAACGCCGACTA-3´ and *c-Fos*-reverse 5´- TGGCACTAGAGACGGACAGAT-3´, nuclear factor of activated T cells, cytoplasmic 1 (*NFATc1*)-forward 5´- GCCTTTTGCGAGCAGTATCTG-3´ and *NFATc1*-reverse 5´- GCTGCACCTCGATCCGAAG-3´, *GAPDH*-forward 5´- AGGTCGGTGTGAACGGATTTG-3´ and *GAPDH*-reverse 5´- TGTAGACCATGTAGTTGAGGTCA -3´. The relative mRNA expression levels were calculated using the 2^−ΔΔCt^ method, and GAPDH was measured as an internal control.

### Bone resorption assay

RAW264.7 cells at a density of 8×10^3^ cells/well were plated on bovine bone slides in 96‐well plates, and cultured in an osteoclast induction medium. On day 7, bone slices were washed 3 times for 5 mins with 40 Hz ultrasonic and fixed in glutaraldehyde 5%. After gradient dehydration, drying and gold-coating, the bovine bone slices were observed under a scanning electron microscope (SEM; Hitachi S-3400N, Tokyo, Japan) at an accelerating voltage of 15 kV to observe and record bone resorption pits. The bone resorption areas were quantified by the Image-Pro Plus software.

### Statistical analysis

Statistical analysis was performed using SPSS 25.0 software. The data are expressed as mean ± standard deviation (SD). Two-group comparisons were conducted using the Students t-test, multiple-group comparisons were conducted using one-way ANOVA, and any two-group comparisons were conducted by SNK-q test. Differences were considered to be statistically significant if *P* < 0.05.

## Results

### Effect of RANKL concentrations on osteoclastogenesis

**Fig 1:**
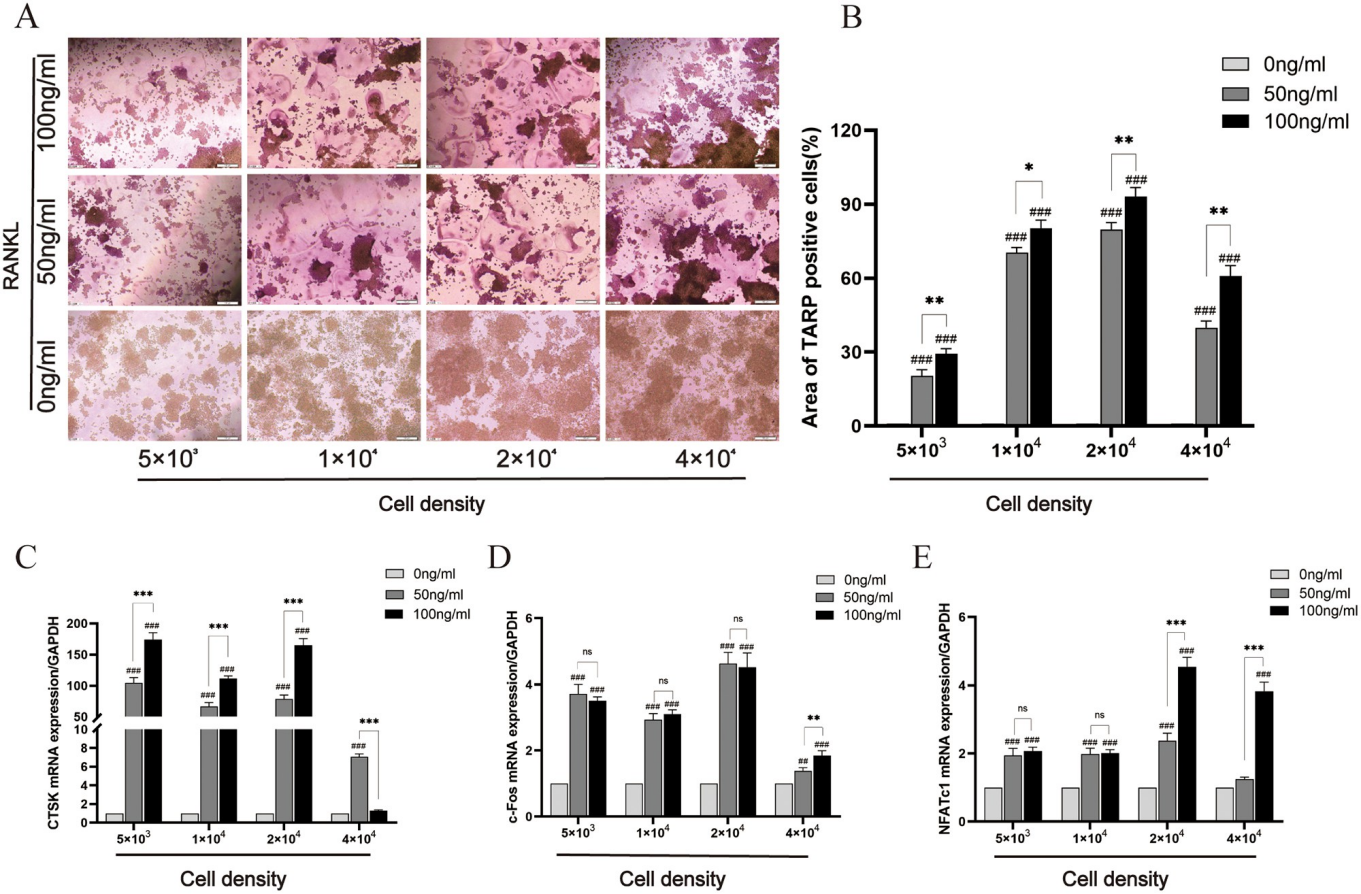
Effects of different concentrations of RANKL alone on osteoclast differentiation in RAW264.7 cells. RANKL (50 ng/ml and 100 ng/ml) induced differentiation of osteoclasts from RAW264.7 cells for 3 days or 5 days. (A) multinucleated osteoclasts were observed by light microscopy on day 5, original magnification of ×200 (Scale bar = 200 μm). (B) TRAP-positive cells area was analyzed and quantified. The expressions of CTSK (C), c-Fos (D) and NFATc1 (E) were tested by qPCR on day 3. Data are presented as mean ± SD (n = 3). #*P*<0.05, ##*P*<0.01, ###*P*<0.001 vs. 0ng/ml RANKL group; *P<0.05, **P<0.01, ***P<0.001 vs. 50ng/ml RANKL group; ns, no significance.

To demonstrate osteoclastogenesis and its activity, TRAP staining and qPCR were used. The TRAP staining results demonstrated that RANKL was an essential cytokine for osteoclastogenesis, and areas of TRAP-positive cells in the presence of 50 ng/ml RANKL was significantly fewer than in the presence of 100 ng/ml RANKL (Fig 1A and 1B). qPCR was used to detect osteoclasts marker genes. The results revealed RANKL treatment could increase CTSK gene expression by 50 to 200 times compared with the untreated group, except for a density of 4×10^4^ cells/well. There was a statistically significant difference in the treatment groups with different concentrations of RANKL (Fig 1C). In addition, our results showed that RANKL stimulated the expression of c-Fos and NFATc1, and there was no statistical difference between RANKL treatment groups when cell densities were 5×10^3^ cells/well and 1×10^4^ cells/well (Fig 1D and 1E). These results indicated that the increase of RANKL concentration increased the formation of multinucleated cells and osteoclast activity.

### Effect of M-CSF on osteoclastogenesis

**Fig 2:**
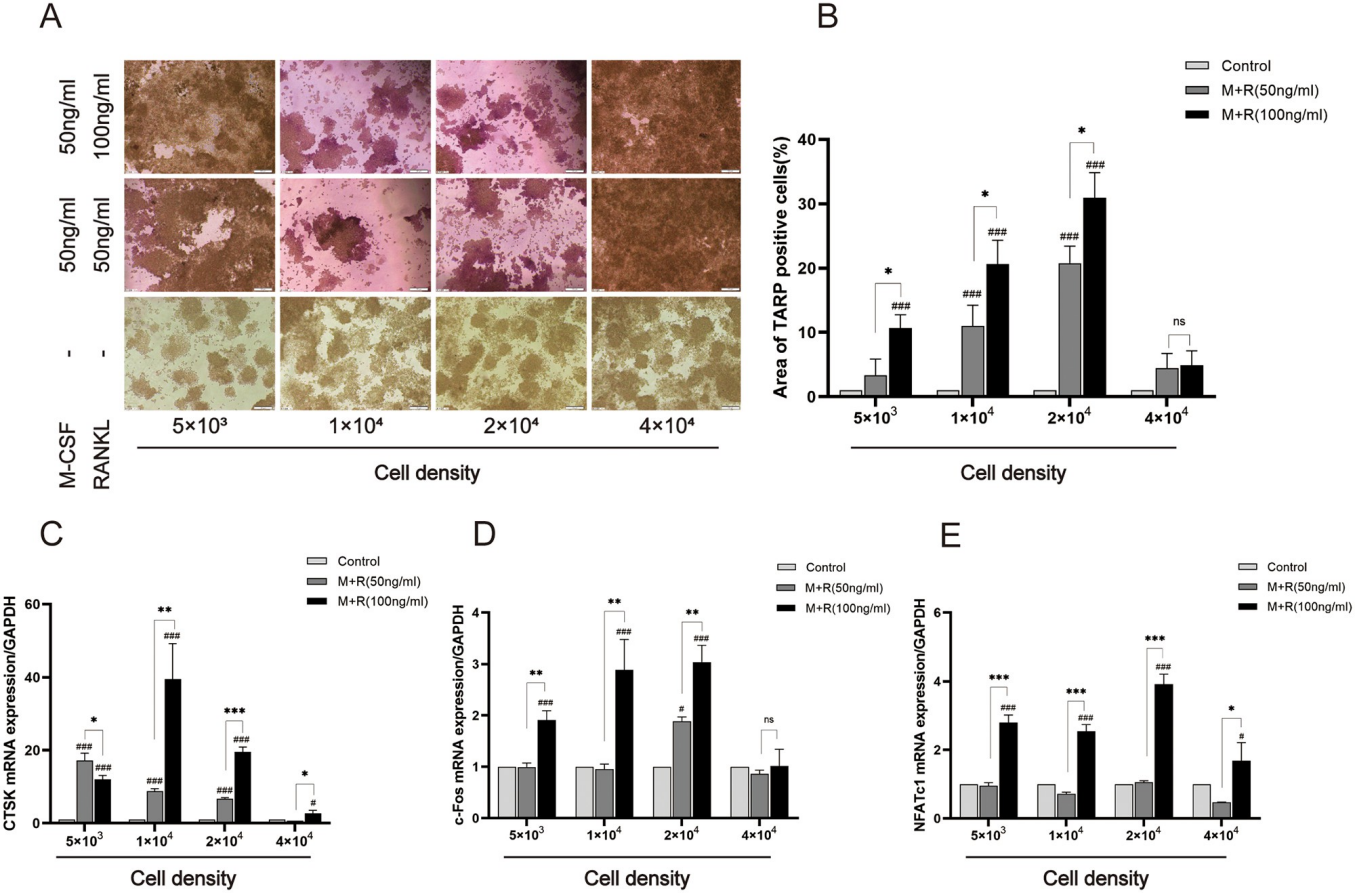
Effects of M-CSF on the differentiation of RAW264.7 cells into osteoclasts. RANKL (50 ng/ml and 100 ng/ml) plus 50 ng/ml M-CSF induced differentiation of osteoclasts from RAW264.7 cells for 3 days or 5 days. (A) multinucleated osteoclasts were observed by light microscopy on day 5, original magnification of ×200 (Scale bar = 200 μm). (B) TRAP-positive cells area was analyzed and quantified. The expressions of CTSK (C), c-Fos (D) and NFATc1 (E) were tested by qPCR on day 3. Data are presented as mean ± SD (n = 3). #*P*<0.05, ##*P*<0.01, ###*P*<0.001 vs. Control group (M-CSF-, RANKL-); *P<0.05, **P<0.01, ***P<0.001 vs. M+R(50ng/ml) group (50ng/ml M-CSF, 50ng/ml RANKL); ns, no significance.

Although recombinant M-CSF is required for the generation of osteoclasts from bone marrow-derived macrophages, it is not essential for the induction of RAW264.7-osteoclasts [17–19]. TRAP staining showed that after treatment with M-CSF, the number of monocytes increased significantly and the formation rate of multinucleated osteoclasts decreased significantly (Fig 2A and 2B). qPCR results showed that the osteoclast-specific gene of CTSK was significantly inhibited by M-CSF (Fig 2C). Transcription factors c-Fos and NFATc1 are crucial in osteoclast differentiation [20]. qPCR results showed treatment with 50 ng/ml RANKL plus M-CSF did not affect c-Fos and NFATc1 expression, but c-Fos and NFATc1 expression were similar to treatments with RANKL alone and 100 ng/ml RANKL plus M-CSF (Fig 2D and 2E). These results indicated that the cells stimulated by M-CSF tended towards early differentiation, but were less mature osteoclasts.

### Effect of RANKL or M-CSF on pit formation

**Fig 3:**
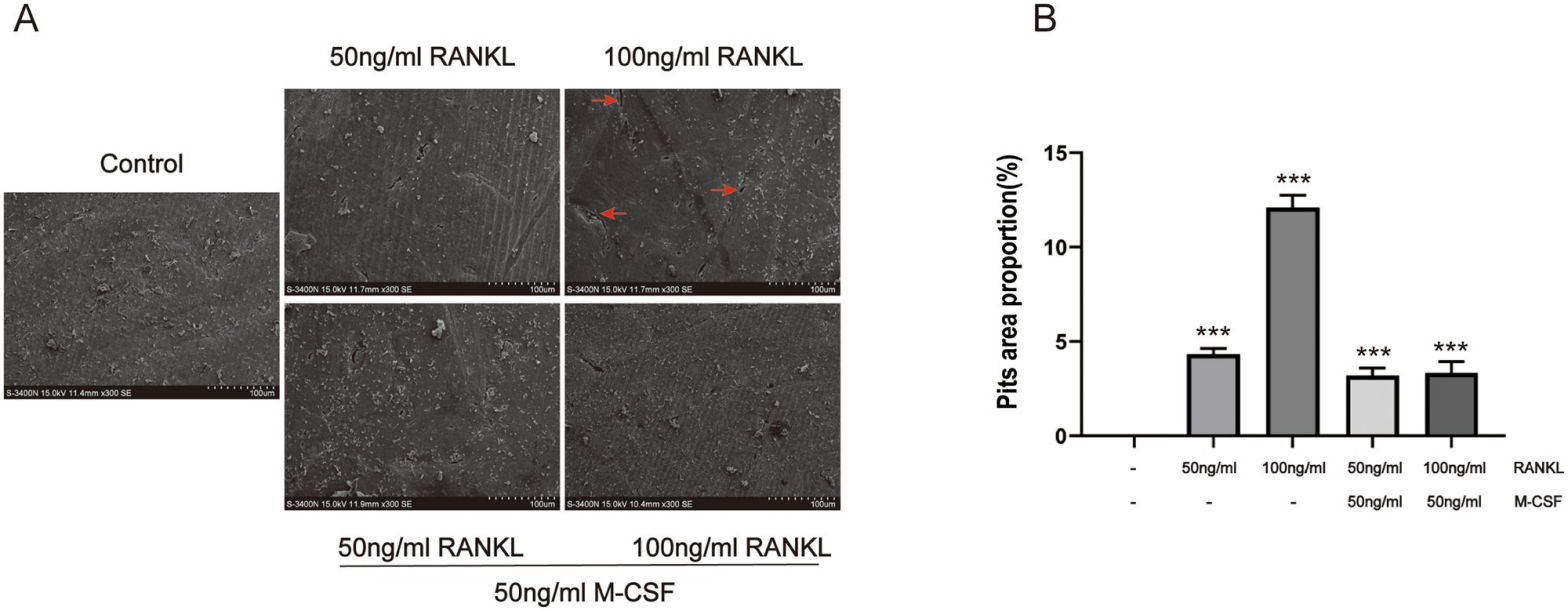
Effects of different induction conditions on bone resorption pits. RAW264.7 cells were plated on bovine bone slides with different concentrations of RANKL with or without M-CSF for 7 days. (A) Bone resorption pits were detected by SEM on day 7, original magnification of ×300 (Scale bar = 100 μm), red arrow indicates bone resorption pits. (B) Quantitative analysis of bone resorption pit area. Data are presented as mean ± SD (n = 3). Statistically significant (***P<0.001).

Although RANKL or M-CSF have the ability to stimulate or inhibit osteoclastogenesis, it is unknown whether these cells possess osteoclast function. To determine osteoclast functionality, we performed an evaluation of pit formation by SEM. The analysis revealed that the fiber structure of bone slice in the untreated group was clear and the surface was flat. After adding RANKL, varying degrees of fiber structure damage were observed in the bone slices, and the surface of bone slices was uneven with bone resorption pits. Treatment with 100 ng/ml RANKL alone resulted in a high number and deep bone resorption pits compared with other treatments (Fig 3). These findings demonstrated that osteoclasts were capable of bone resorption and that the greater the number of osteoclasts, the more bone lacuna produced.

### Effect of cell density on osteoclastogenesis

**Fig 4:**
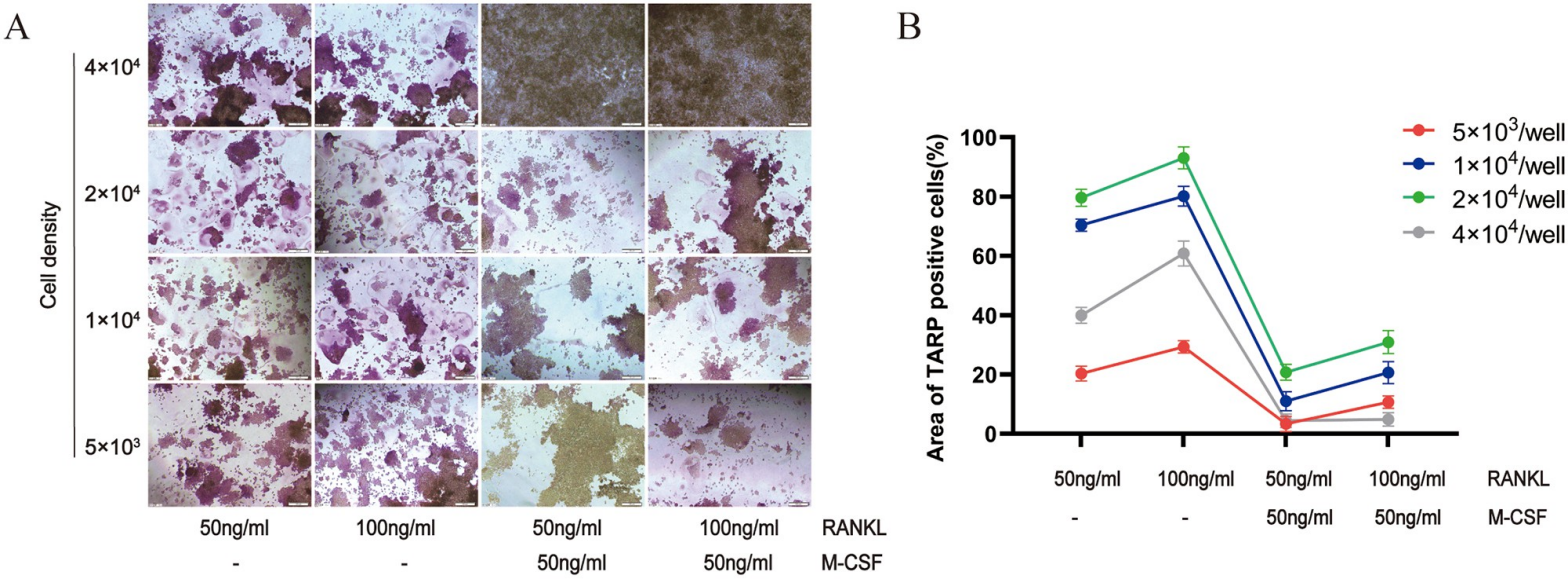
Effects of different cell densities on differentiation of RAW264.7 cells into osteoclasts. (A) Multinucleated cells were stained by TRAP staining and observed under light microscopy, original magnification of ×200 (Scale bar = 200 μm). (B) TRAP-positive cells area was analyzed and quantified.

Cell density is an important parameter affecting differentiation. To determine the effect of cell density on cell differentiation, TRAP staining was used. TRAP staining showed that from 5×10^3^ cells/well to 2×10^4^ cells/well, the induction efficiency of RAW264.7-osteoclasts increased with the increase of cell density. However, high cell density (4×10^4^ cells/well) promoted the superimposed growth of cells and reduced the formation of osteoclasts (Fig 4). These findings revealed that excessive or low cell density affects the induction efficiency and that cell differentiation into mature osteoclasts requires a suitable cell density and adequate growth space.

## Discussion

Hsu et al. first reported in 1999 that recombinant RANKL factor could induce RAW264.7 cells into osteoclasts, which are highly homogeneous with bone marrow-derived osteoclasts [21]. Since then, RAW264.7 cells have been widely used to research osteoclast-mediated diseases ranging from rheumatoid arthritis and osteoporosis to periodontitis. However, because of the different experimental conditions, cell phenotype and induction methods used, the optimal induction concentration of RANKL reported in the literature ranges from 25 to 100 ng/ml, and there is no consensus on whether other recombinant factors are needed for co-culture [22–24]. Therefore, we evaluated the induction efficiency of different concentrations of RANKL, the effects of M-CSF on RANKL-induced osteoclastogenesis in RAW264.7 cells and the effect of cell density on the induction efficiency.

The results showed that an increase in RANKL promoted osteoclast formation under constant cell densities, indicating that RANKL was essential in the differentiation of RAW264.7 cells into osteoclasts, which provides a reference for subsequent experimental research. Some studies have also pointed out that RANKL at the concentration of 10 to 20 ng/ml can only cause the aggregation of RAW264.7 cells without osteoclasts formation [25]. The induction efficiency of RANKL on RAW264.7 plateaus at a certain concentration, which is not completely proportional to the concentration. There was no significant difference in osteoclast formation induced by RANKL at concentrations equal to or higher than 100 ng/ml [25]. M-CSF is indispensable for the proliferation and survival of osteoclast precursors through extracellular-regulated kinase and protein kinase B [26]. After validating the key role of RANKL, we investigated whether M-CSF plays an essential role in the formation of osteoclasts. While maintaining constant cell density and RANKL concentration, we found that M-CSF supplementation can reduce osteoclastogenesis and bone-resorption activities. This may be because M-CSF supplementation increased the number of monocytes, thereby reducing the formation of multinucleated cells (Fig 2A). Consistent with previous reports, M-CSF is responsible for cellular proliferation, while RANKL mainly promotes cellular differentiation [27]. Induction time and cell density are two key factors related to the differentiation of RAW264.7 cells [28]. The effects of cell density were studied while the induction factor and the induction time remained unchanged. The RAW264.7 cells inoculated in 24-well plates at 5×10^3^ cells/well were not conducive to the migration and fusion of osteoclast precursor cells, while the 4×10^4^ cells/well concentration was too high to form mature osteoclasts with vesicle structure. The highest induction efficiency was observed at a density of 2×10^4^ cells/well.

In addition, osteoclast differentiation by using RAW264.7 cells is also regulated by additional factors like L-glutamine concentration and different cell passages. 4–6 mM L-glutamine in the cell culture medium reduced RANKL-induced osteoclast formation in RAW264.7 cells. In contrast, 1–2 mM L-glutamine had no effect on the induction efficiency of RANKL [16]. Based on the gene expression, RAW264.7 cells of 5–50 passages can be divided into increasing type, stable type and fluctuating type. Moreover, the gene function may vary according to the cell passage [29]. This undoubtedly provides valuable insight and is a challenge to overcome in the culture and induction of RAW264.7 cells.

## Conclusion

In conclusion, this paper explores the effects of different concentrations of RANKL for osteoclast differentiation and confirms that co-culture with M-CSF and RANKL is not essential for osteoclast formation. Furthermore, the requirement of cell density in osteoclast differentiation is described, providing an accurate reference method for osteoclast differentiation of RAW264.7 cells.

## Supporting information

S1 File This supporting information file includes the values used to generate prism plots for all the figures. (XLSX)
